# Intensive insulin therapy in critically ill children: impact on blood glucose dynamics and its relation with mortality

**DOI:** 10.1186/cc12401

**Published:** 2013-03-19

**Authors:** M Van Tornout, M Gielen, T Van Herpe, D Vlasselaers, L Desmet, I Vanhorebeek, G Van den Berghe, D Mesotten

**Affiliations:** 1 University Hospitals Leuven, Belgium

## Introduction

A large RCT showed that tight glucose control (TGC), targeting age-adjusted normal fasting blood glucose levels with insulin infusion, decreased morbidity and mortality in critically children [1]. However, the incidence of hypoglycemia increased substantially in the TGC group. We aimed to assess the effect of TGC on the three domains of blood glucose dynamics (hyperglycemia, hypoglycemia and blood glucose variability) and their independent association with mortality in the pediatric ICU.

## Methods

This is a preplanned substudy of a published RCT in one 10-bed pediatric ICU. Seven hundred patients (age 1 to 16 years), admitted to the PICU between October 2004 and December 2007, were randomized to either TGC (50 to 803 mg/dl in infants, 70 to 1003 mg/dl in children) or to the usual care tolerating hyperglycemia up to 2153 mg/ dl (UC). Patients with at least two arterial blood glucose measurements were included (UC *n *= 349; TGC *n *= 348).

## Results

Mean blood glucose levels were lowered from 143 ± 34 mg/dl in the UC group to 104 ± 24 mg/dl (*P *0.0001). The median number of samples per patient did not differ between UC (21 (13 to 48)) and TGC (22 (15 to 49)). TGC lowered the hyperglycemic index, a marker of hyperglycemia over time, from median 44 (IQR 29 to 66) mg/dl in UC to 12 (7 to 23) mg/dl, as well as the glycemic penalty index, an aggregate measure of dysglycemia, from median 44 (32 to 58) in UC to 22 (16 to 30) (both *P *0.0001). Despite frequent hypoglycemia <40 mg/dl (1.4% in UC, 25.0% in TGC, *P *0.0001), TGC did not result in increased blood glucose variability, as reflected by the standard deviation of the blood glucose time series (TGC: 36 (24 to 50) mg/dl, UC 34 (26 to 48) mg/dl, *P *= 0.82). In multivariable logistic regression analysis, adjusting for baseline risk factors (age, severity of illness, malignancy, type of admission), hypoglycemia (OR = 1.6, 95% CI = 0.19 to 2.22) and blood glucose variability (OR = 1.0, 95% CI = 0.97 to 1.02, per mg/dl) were not associated with PICU mortality. In contrast, hyperglycemic index (OR = 1.02, 95% CI = 1.01 to 1.04, per mg/dl) was independently associated with mortality and its lowering statistically explained the outcome benefit of the intervention.

## Conclusion

TGC substantially reduced the hyperglycemic index, at the expense of increased hypoglycemia incidence, but without affecting blood glucose variability. Statistical analysis suggested strictly avoiding hyperglycemia in order to bring about outcome benefit with insulin in critically ill children.

## References

[B1] Vlasselaers D Lancet 2009 373 547 556 10.1016/S0140-6736(09)60044-1 19176240

